# The latest research progress of ligustilide in the prevention and treatment of central nervous system disorders

**DOI:** 10.3389/fphar.2026.1843537

**Published:** 2026-06-22

**Authors:** Jin Han, Yongkang Sun, Yanbo Song, Yijun Wu, Xinzhi Wang

**Affiliations:** 1 Encephalopathy Center, The First Affiliated Hospital of Henan University of Chinese Medicine, Zhengzhou, China; 2 The First Clinical Medical School of Henan University of Chinese Medicine, Zhengzhou, China; 3 Collaborative Innovation Center of Prevention and Treatment of Major Diseases by Chinese and Western Medicine, Zhengzhou, China; 4 Institute of Encephalopathy, Henan Academy of Chinese Medicine, Zhengzhou, China

**Keywords:** active ingredients of Chinese medicine, central nervous system, ligustilide, mechanism of action, research progress

## Abstract

**Background:**

Ligustilide (LIG), a natural phthalide compound mainly isolated from Angelica sinensis and Ligusticum chuanxiong, has attracted increasing attention because of its diverse pharmacological activities, including anti-inflammatory, antioxidant, anti-apoptotic, and neuroprotective effects. Emerging studies suggest that LIG may have therapeutic relevance in central nervous system (CNS) disorders.

**Purpose:**

This review systematically summarizes the pharmacological effects, molecular mechanisms, pharmacokinetic characteristics, metabolism, safety profile, and therapeutic potential of LIG in CNS disorders.

**Methods:**

Relevant studies published up to 26 October 2025 were retrieved from PubMed, Web of Science, and Scopus using keywords related to ligustilide, central nervous system disorders, pharmacokinetics, metabolism, and toxicity. After removing duplicate records and excluding reviews, editorials, and irrelevant articles, 55 eligible original studies were included in this review.

**Results:**

Current evidence indicates that LIG exerts neuroprotective effects in multiple CNS disorders, including ischemic stroke, cerebral ischemia–reperfusion injury, vascular dementia, Alzheimer’s disease, Parkinson’s disease, traumatic brain injury, and anxiety disorders. Its mechanisms mainly involve modulation of PI3K/Akt, MAPK, NF-κB, Nrf2/ARE, AMPK, and other signaling pathways, leading to reduced oxidative stress, inflammation, apoptosis, and mitochondrial dysfunction. In addition, available studies suggest that LIG can cross the blood–brain barrier and shows relatively favorable safety in preclinical models.

**Conclusion:**

LIG demonstrates broad neuroprotective potential in preclinical studies and may represent a promising candidate for CNS disease intervention. However, its poor chemical stability, low oral bioavailability, limited toxicity evaluation, and lack of clinical evidence remain major challenges for translational application. Further studies are required to optimize delivery strategies and validate its efficacy and safety in clinical settings.

## Introduction

Central nervous system (CNS) disorders, including ischemic stroke, Alzheimer’s disease (AD), Parkinson’s disease (PD), vascular dementia (VaD), and traumatic brain injury (TBI), are major causes of disability and mortality worldwide. These disorders are characterized by complex pathological processes, including neuroinflammation, oxidative stress, mitochondrial dysfunction, and neuronal apoptosis, which ultimately contribute to progressive neurological impairment and cognitive decline (Verkhratsky et al., 2023; Feigin et al., 2020). Current treatment strategies remain limited and primarily focus on symptomatic management. Effective neuroprotective agents capable of delaying disease progression are still lacking. Natural products have attracted increasing attention in the development of therapies for CNS disorders because of their diverse biological activities and favorable biocompatibility. Ligustilide (LIG), a major phthalide compound isolated from Angelica sinensis and Ligusticum chuanxiong, exhibits multiple pharmacological properties, including anti-inflammatory, antioxidant, anti-apoptotic, and neuroprotective effects (Wu et al., 2019; Mao et al., 2022). Increasing evidence suggests that LIG exerts protective effects in various CNS disorders through modulation of multiple signaling pathways, including PI3K/Akt, NF-κB, MAPK, AMPK, and Nrf2/ARE pathways. These effects are closely related to the regulation of neuroinflammation, oxidative stress, mitochondrial homeostasis, and neuronal survival.

## Methods

**FIGURE 1:**
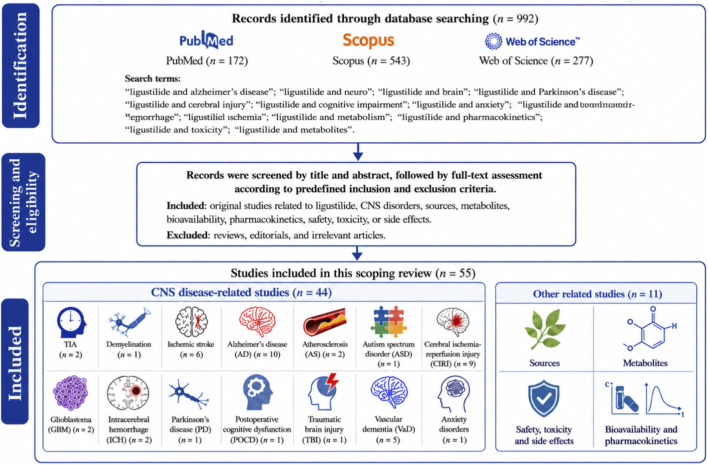
The PRISMA flowchart for literature search, screening, eligibility assessment and inclusion of studies on the treatment of central nervous system disorders with LIG.

Relevant studies published up to 26 October 2025 were retrieved from PubMed, Web of Science, and Scopus databases according to the PRISMA guidelines. Detailed search strategies for each database are provided in the Supplementary Material. Studies investigating the pharmacological effects, mechanisms, pharmacokinetics, metabolism, toxicity, and delivery strategies of ligustilide in CNS disorders were considered eligible. Reviews, editorials, conference abstracts, duplicate publications, and studies unrelated to CNS diseases or ligustilide were excluded. Literature screening was independently performed based on titles, abstracts, and full-text evaluation to ensure relevance to the topic. The literature screening process is shown in Figure 1.

## Overview of LIG

### The source of LIG

LIG is one of the major bioactive phthalide compounds isolated from Angelica sinensis and Ligusticum chuanxiong, two traditional medicinal herbs belonging to the Apiaceae family that have long been used in the treatment of cerebrovascular and neurological disorders (Wang Y et al., 2025a). Previous studies have demonstrated that the content of LIG varies according to species, geographical origin, cultivation environment, and processing methods (Zhao et al., 2003; Donkor et al., 2016). In addition, the distribution of LIG within medicinal plants is tissue-specific, with relatively higher levels observed in rhizomes (Zheng et al., 2021). From the perspective of traditional Chinese medicine, Ligusticum chuanxiong is a key herb for promoting blood circulation, regulating qi, dispelling wind and relieving pain. It has been used in clinical practice for thousands of years. Angelica sinensis is sweet, warm, moist in nature, pungent and warm in circulation. It is traditionally used for tonifying blood, promoting blood circulation, regulating menstruation and relieving pain, as well as moistening the intestines and promoting defecation. Both are important medicinal herbs in clinical prescriptions for promoting blood circulation and removing blood stasis (Yu et al., 2008). Traditional processing and extraction procedures may also influence the stability and pharmacological activity of LIG. Experimental studies have shown that different processing conditions, including alcohol treatment and decoction duration, can alter the composition and biological activity of Ligusticum chuanxiong extracts (Ning et al., 2019; Zhang et al., 2019). These findings suggest that standardization of cultivation, extraction, and processing procedures may be important for improving the stability and therapeutic potential of LIG. Collectively, current evidence indicates that LIG is an important natural compound with considerable pharmacological value and promising therapeutic potential in CNS disorders.

### Chemical characteristics and stability of LIG

**FIGURE 2:**
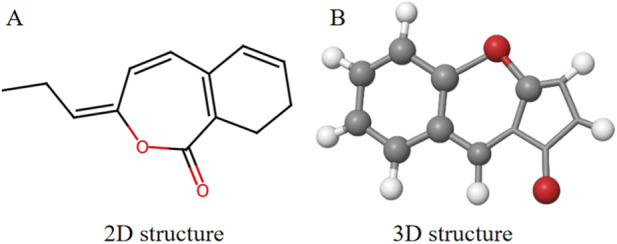
Chemical structure of LIG (A:2D structure of ligustilide; B:3D structure of ligustilide). The image is sourced from https://pubchem.ncbi.nlm.nih.gov/compound/531902.

LIG is a representative phthalide compound isolated from Angelica sinensis and Ligusticum chuanxiong, with the molecular formula C12H14O2 and a relative molecular mass of 190.24 (Figure 2). It is a slightly yellow oily compound with poor aqueous solubility but good solubility in organic solvents such as methanol, ethanol, ether, ethyl acetate, and petroleum ether. These physicochemical properties are closely related to its absorption behavior, formulation development, and pharmaceutical applicability. However, LIG has relatively poor chemical stability. Its unsaturated phthalide skeleton, butenyl side chain, and lactone bond make it susceptible to oxidation, isomerization, hydrolysis, and degradation. Previous studies have shown that alkaline conditions, especially when the pH is higher than 8.0, can accelerate lactone bond hydrolysis and promote LIG degradation (Gui et al., 2013). Temperature is another important factor affecting its stability, with higher temperatures leading to faster degradation (Xie et al., 2020). In addition, light exposure may further reduce the structural stability of LIG, whereas sealed, light-protected, and low-temperature storage conditions can improve its stability (Cui et al., 2006).

### The bioavailability and pharmacokinetics of LIG

The pharmacokinetic characteristics of LIG play an important role in determining its therapeutic efficacy and clinical translational potential. Previous studies have demonstrated that LIG exhibits relatively low oral bioavailability because of its poor aqueous solubility, rapid metabolism, and extensive first-pass elimination. Experimental evidence further suggests that LIG follows a dose-dependent pharmacokinetic profile and generally conforms to a two-compartment model with first-order absorption *in vivo* (Zhang et al., 2014a). Systematic pharmacokinetic studies have shown that the oral bioavailability of LIG is relatively limited, although measurable plasma exposure can still be achieved after administration (Yan et al., 2008). Increasing the dosage significantly enhances systemic exposure, as reflected by elevated area under the concentration-time curve (AUC) values (Zhang et al., 2020). In addition, tissue distribution studies based on high-performance liquid chromatography have demonstrated that the liver is the major organ responsible for LIG metabolism and elimination (Qian et al., 2008). Importantly, LIG is capable of penetrating the blood-brain barrier and exhibits preferential distribution in the cerebellum and cerebrum, providing an important pharmacokinetic basis for its neuroprotective effects in CNS disorders (Wu et al., 2019; Li et al., 2017a). Relatively high concentrations have also been observed in the spleen and kidney, indicating broad tissue distribution characteristics. Overall, although LIG possesses promising brain-targeting potential, its poor oral bioavailability and rapid metabolic clearance remain major limitations for clinical translation. Therefore, optimization of delivery systems and formulation strategies may be essential for improving its pharmacokinetic properties and therapeutic efficacy.

## Metabolites of LIG

LIG is mainly metabolized in the liver through multiple biotransformation reactions, including oxidation, reduction, and hydrolysis, resulting in the formation of butenylphthalide and carboxylic acid compounds (Duan et al., 2018). With the development of advanced analytical techniques, increasing evidence has clarified the metabolic characteristics of LIG *in vivo*. Using ultra-performance liquid chromatography coupled with high-resolution mass spectrometry (UPLC-HRMS), Tao et al. demonstrated that Senkyunolide I is one of the major metabolites of LIG in human hepatocytes and reported species-dependent differences in its metabolic half-life between rats and humans (Tao et al., 2020). In addition, Ding et al. identified four characteristic metabolites of LIG in rat urine by high-performance liquid chromatography analysis, including 6,7-dihydro-6,7-dihydroxyligustilide (M1), 3,5-dihydroxy-3-butylphthalide (M2), 6,7-dihydro-6-hydroxy-7-sulfonatomethyl ligustilide (M3), and 5-hydroxy-3-butylidenephthalide (M4) (Ding et al., 2008). These findings provide important evidence for understanding the metabolic pathways, pharmacokinetic behavior, and bioactive transformation mechanisms of LIG *in vivo*.

## Safety, toxicity and side effects of LIG

Although LIG is a natural bioactive compound isolated from traditional medicinal herbs such as Angelica sinensis and Ligusticum chuanxiong, and is generally considered to exhibit favorable biocompatibility and relatively low toxicity, excessive exposure may still induce adverse biological effects. Therefore, its safety profile and potential toxicological risks have attracted increasing attention. Acute toxicity studies have demonstrated that LIG possesses relatively low toxicity under conventional experimental conditions. Zhang et al. reported that the oral LD50 of LIG in mice was 7.23 g/kg, whereas the intraperitoneal LD50 was 2.25 g/kg, both of which were substantially higher than the conventional therapeutic dose range, indicating relatively favorable acute safety (Zhang et al., 2012). However, mild skin irritation associated with LIG has also been reported, suggesting that topical administration may require additional safety evaluation. The toxic effects of LIG appear to be dose- and time-dependent. Experimental studies have shown that LIG may induce reactive oxygen species (ROS) generation and transient glutathione depletion under certain conditions, thereby triggering oxidative stress-related cellular injury (Qi et al., 2012a). In addition, potential interactions between LIG and dopaminergic agents have been reported. Co-administration of LIG with dopamine was shown to further increase ROS production and aggravate oxidative stress-induced cellular damage, indicating that combined use with dopaminergic drugs may require careful evaluation (Qi et al., 2012b). Overall, current evidence suggests that LIG exhibits relatively favorable safety in preclinical studies. However, its long-term toxicity, pharmacological interactions, and clinical safety profile remain insufficiently investigated, and further systematic studies are still required before clinical application can be fully considered.

## The pharmacological activity and mechanism of action of LIG in the treatment of CNS disorders

A substantial body of evidence has demonstrated that LIG exerts preventive and therapeutic effects on a variety of CNS disorders, characterized by multi-target and multi-pathway actions. To further elucidate its roles in CNS disorders, numerous experimental studies have been conducted to investigate its pharmacological activities and underlying mechanisms. Table 1 summarizes the experimental designs, pharmacological effects, and mechanisms of LIG in the context of CNS disorders.

**TABLE 1:** Mechanisms of action of LIG in the CNSD.

CNSD		Study type	Experimental models	Main effect of LIG	Main mechanisms/Pathways	Ref
Ischemic stroke	IS	Combined	C57BL/6J mice; HEK293 cells	Reduced neuroinflammation, oxidative stress, and neuronal loss	RIG-I/NF-κB and AKT/FOXO1 pathways	Long et al. (2018)
	IS	*In vitro*	Recombinant mouse Prxs	Reduced inflammatory responses	TLR4/NF-κB signaling pathway	Zhao et al. (2016)
	IS	*In vivo*	MCAO rat models	Reduced BBB injury, oxidative stress, and brain edema	Nrf2/HSP70 signaling pathway	Li et al. (2017b)
	IS	*In vivo*	C57/BL6 mice	Promoted angiogenesis and neurological recovery	Increased of VEGF and eNOS activation	Ren et al. (2020)
	IS	Combined	HT22 cells; MCAO/R mice	Improved mitochondrial dysfunction and inhibited apoptosis	AMPK signaling pathway	Wu et al. (2022)
Cerebral ischaemia-reperfusion injury	CIRI	*In vivo* using MCAO rat models		Improved neurological function and attenuated neuroinflammation, oxidative stress, and neuronal injury	TLR4/PRX6 and Nrf2/HO-1 signaling pathways	Kuang et al. (2014); Peng et al. (2013)
	CIRI	Combined	MCAO/R rats; OGD/R HT22 cells	Promoted mitophagy and reduced oxidative stress	PINK1/PARKIN signaling pathway	Mao et al. (2022)
	CIRI	*In vitro*	OGD/R-treated PC12 cells	Promoted autophagy and reduced neuronal apoptosis	LKB1-AMPK-mTOR signaling pathway	Zhao et al. (2020)
	CIRI	Combined	OGD/R neurons; cerebral I/R rats	Reduced oxidative stress and neuronal apoptosis	PI3K/AKT signaling pathway	Wu et al. (2020)
	CIRI	*In vivo*	2VO rat models	Reduced neuronal apoptosis and hypoperfusion injury	Reduced Caspase-3; increased the number of Nissl body and NeuN-positive cells and improved the MAP-2positive structures	Wang et al. (2016)
	CIRI	Combined	MCAO/R mice BV-2 cells	Reduced pyroptosis and neuroinflammation	NRF2 signaling pathway	Wang et al. (2025a)
	CIRI	Combined	Ischemia-reperfusion and OGD models	Reduced neuronal apoptosis	ERK signaling pathway	Wu et al. (2011)
	CIRI	Combined	Rat stroke model; PC12 cells	Reduced apoptotic and necrotic cell death	MAPK/HSP70 signaling pathway	Yu et al. (2015)
Transient ischaemic attacks	TIA	*In vivo*	FCI mice	Reduced oxidative stress and neuronal apoptosis	Reduced MDA, Bax and caspase-3; increased expression of GSH-PX, SOD and Bcl-2	Kuang et al. (2009)
Intracerebral hemorrhage	ICH	*In vivo*	Autologous blood-induced ICH models	Reduced neuroinflammation	Prx1/TLR4/NF-κB signaling pathway	Han et al. (2018)
	ICH	*In vivo*	Double hemorrhage models	Reduced neuronal apoptosis and brain injury	Downregulated Bax, p53 and cleaved caspase-3, and upregulated Bcl-2	Chen et al. (2011)
Geriatric traumatic brain injury	TBI	*In vivo*	TBI mouse models	Reduced neuroinflammation and neuronal apoptosis; promoted autophagy and M2 microglial polarization	Downregulated the expression of p62, Bax, iNOS, IL-6, TNF-α, Iba1, CD68, ROS levels, and cleaved caspase-3; upregulated the LC3II/LC3I ratio, Bcl-2, and Arg1	Wu et al. (2025)
Vascular dementia	VaD	*In vivo* using 2VO models		Improvement of cholinergic dysfunction and attenuation of oxidative stress, neuronal apoptosis, and astrocyte proliferation	Regulation of oxidative stress-, cholinergic dysfunction-, and apoptosis-related pathways	Kuang et al. (2008); Feng et al. (2012)
	VaD	*In vivo* using VaD rats		Reduced oxidative stress and neuronal apoptosis	AMPK/SIRT1 signaling pathway and SIRT1/IRE1 α/XBP1s/CHOP pathway	Peng et al. (2022a)
	VaD	*In vivo*	Bilateral carotid artery occlusion rats	Promoted neurogenesis and neuronal repair	Upregulated BDNF,p- CREB, GABA	Xin et al. (2013)
Alzheimer’s disease	AD	*In vivo*	Scopolamine-induced mice	Reduced neuroinflammation and oxidative stress	TLR4/NF-κB signaling pathway	Zhang et al. (2025)
	AD	In vivo using APP/PS1 mice		Improved mitochondrial function; protected synapses; reduced oxidative stress and neuronal apoptosis	PKA/AKAP1 signaling pathway; decreased Drp1, MDA, ROS, and Aβ levels, while increased Mfn1, Mfn2, Opa1, ATP, CCO, SDH, Mn-SOD, PSD-95, synaptophysin, and synapsin-1 levels	He et al. (2024b); Xu et al., (2018)
	AD	*In vivo*	SAMP8 mice	Inhibit apoptosis, oxidative stress and inflammatory responses; improve mitochondrial function	Inhibited P-Drp1 and malondialdehyde levels, while increased Mfn1, Mfn2, P-AMPK, ATP, and superoxide dismutase levels	Zhu et al. (2020)
	AD	*In vivo*	D-galactose-induced aging mice	Inhibited oxidative stress and apoptosis and alleviated neurotoxicity	Decreased MDA,cleaved caspase-3,and GFAP levels; increased Na+-K+-ATP ase and GAP-43 levels	Li et al. (2015)
	AD	*In vitro*	SH-SY5Y cells	Inhibited apoptosis and endoplasmic reticulum stress and promoted autophagy	Decreased LC3B-II/I,P62/SQSTM1,PERK,GRP78,CHOP,Bax, caspase-12,Beclin1,and Atg5 levels, while increased Bcl-2 levels	Gao et al. (2021)
	AD	*In vitro*	SH-SY5Y and PC12 cells	Reduced oxidative stress and neurotoxicity	PI3K/AKT and p38 signaling pathways	Xu et al. (2016)
	AD	*In vivo*	Aβ25-35-induced rats	Inhibited inflammatory responses and alleviated neurotoxicity	Decreased TNF-α,NF-κB	Kuang et al. (2009)
	AD	Combined	APP/PS1 mice; SH-SY5Y cells	Reduced Aβ accumulation and neuronal injury	IGF-1/AKT/MTOR signaling pathway	Kuang et al. (2017)
	AD	Combined	SAMP8 mice; 293T cells	Attenuated oxidative stress; reduced Aβ1–42 accumulation and tau phosphorylation	Decreased MDA, carbonylated proteins, the DNA oxidation product 8-hydroxy-deoxyguanosine, p-IRS-1, p-Akt, Aβ1–42, p-Tau, and SA-β-gal levels; increased Klotho, FoxO1, catalase, and Mn-SOD levels; active IGF-1 signaling	Katonova et al. (2025)
Postoperative cognitive dysfunction	POCD	*In vivo*	tGCI/R mice	Promoted neurogenesis, proliferation,and differentiation	Increased Syp,PSD-95,GAP-43,Syn-IIa,and ERK1/2 levels	Sun et al. (2021)
Parkinson’s disease	PD	*In vivo*	MPTP-induced PD mice	Inhibited inflammation, apoptosis, and oxidative stress; modulated microglial polarization	NRF2/TRXR signaling pathway	Peng et al. (2024)
Glioblastoma multiforme	GBM	*In vitro*	T98G cells	Regulate cell migration	Reduce T98G cell migration ability; inhibit ERK-MAP kinase, RhoA,Cdc42,Rac1,TNF-α	Yin et al. (2013)
Atherosclerosis	AS	Combined	EA.hy926 cells; LDLR^−/−^ mice	Reduced oxidative stress and endothelial dysfunction	NRF2/ARE signaling pathway	Zhu et al. (2019)
	AS	*In vivo*	ApoE^−/−^ mice	Improved lipid metabolism and intestinal barrier integrity	Gut microbiota- and cannabinoid receptor 2-related signaling pathways	Liu et al. (2024)
Autism spectrum disorder	ASD	*In vivo*	VPA-induced and BTBR mice	Improved autism-like behaviors and reduced ferritinophagy	ULK1/NCOA4 signaling pathway	Zhou et al. (2024)
Demyelination		*In vivo*	dMCAO models	Reduced neuroinflammation and promoted remyelination	AIM2/CASPASE-1 signaling pathway	Liang et al. (2025)
Anxiety disorders		*In vivo*	ALAN-exposed mice	Reduced neuroinflammation, mitophagy, and synaptic injury	PI3K/AKT/MTOR signaling pathway	Gao et al. (2025)

## The therapeutic effects of LIG in ischemic stroke

Ischemic stroke is characterized by cerebral blood flow obstruction and subsequent neuronal injury involving neuroinflammation, oxidative stress, mitochondrial dysfunction, and apoptosis (Hurford et al., 2020). In recent years, increasing evidence has demonstrated that LIG exerts significant neuroprotective effects against ischemic stroke through multiple mechanisms. Long et al. reported that LIG inhibited activation of the RIG-I/NF-κB p65 and Akt/FoxO1 signaling pathways, thereby suppressing neuroinflammation and oxidative stress and reducing neuronal loss in the hippocampal CA1 region and caudate-putamen after cerebral ischemia (Long et al., 2018). Zhao et al. further demonstrated that LIG attenuated inflammatory responses induced by peroxiredoxin subtypes through modulation of the TLR4/NF-κB signaling pathway, suggesting that LIG may alleviate macrophage-mediated neuroinflammatory injury following cerebral ischemia (Zhao et al., 2016). To overcome the poor oral bioavailability and instability of LIG, Li et al. developed an intranasal delivery strategy and demonstrated that intranasal administration of Z-ligustilide rapidly penetrated the blood–brain barrier and exerted superior neuroprotective effects compared with oral administration. These effects were associated with activation of the Nrf2 and HSP70 signaling pathways, leading to attenuation of oxidative stress, neuroinflammation, blood-brain barrier disruption, and brain edema (Li et al., 2017a).

In addition, Ren et al. showed that LIG promoted angiogenesis and improved neurological function by increasing vascular endothelial growth factor expression and endothelial nitric oxide synthase (eNOS) activation in ischemic brain tissue (Ren et al., 2020). Wu et al. demonstrated that LIG activated the AMPK signaling pathway and regulated mitochondrial dynamics, thereby improving mitochondrial function, reducing neuronal apoptosis, and alleviating ischemic brain injury (Wu et al., 2022). Furthermore, Chi et al. reported that pretreatment of adipose-derived stem cells with LIG significantly enhanced cell survival and anti-apoptotic capacity after transplantation into thromboembolic stroke mice. LIG-pretreated stem cells also exhibited increased differentiation toward neuronal and vascular endothelial cell phenotypes, suggesting a synergistic therapeutic effect between LIG and stem cell therapy (Chi et al., 2016). A recent meta-analysis of preclinical studies further demonstrated that ligustilide reduced infarct volume and improved neurological deficit scores in ischemic stroke models, providing additional evidence supporting its neuroprotective efficacy (An et al., 2025). Collectively, current evidence indicates that LIG exerts protective effects against ischemic stroke mainly through anti-inflammatory, antioxidant, anti-apoptotic, mitochondrial-protective, and pro-angiogenic mechanisms involving multiple signaling pathways, including NF-κB, Nrf2/HSP70, AMPK, and PI3K/Akt-related pathways.

## The therapeutic effects of LIG in cerebral ischaemia-reperfusion injury

Stroke is one of the leading diseases threatening human health. The primary goal of clinical treatment is to restore cerebral blood perfusion; however, the restoration of blood flow to ischemic brain tissue often triggers a series of “cascade-like” ischemic reactions, which may further aggravate brain injury. This pathological process is known as cerebral ischemia-reperfusion injury (CIRI) (Goncalves et al., 2022). In recent years, LIG, as a bioactive compound with potential neuroprotective properties, has attracted increasing attention for its therapeutic effects and mechanisms in CIRI. Accumulating evidence suggests that LIG exerts neuroprotective effects through multi-target and multi-pathway interactions, primarily involving the regulation of mitophagy, inhibition of oxidative stress, attenuation of neuroinflammation, and reduction of apoptosis.

At the level of signaling pathway regulation, LIG has been shown to modulate multiple pathways, including TLR4/Prx6 (Kuang et al., 2014), Nrf2/HO-1 (Peng et al., 2013), PINK1/Parkin (Kuang et al., 2014), LKB1-AMPK-mTOR (Zhao et al., 2020), PI3K/Akt (Wu et al., 2020) as well as regulation of MAP2 expression (Wang et al., 2016). Through these mechanisms, LIG promotes mitophagy, suppresses oxidative stress and neuroinflammatory responses, and reduces apoptosis, thereby protecting against neuronal injury induced by ischemia-reperfusion. Notably, LIG can also regulate Nrf2 expression and reduce mitochondrial ROS production, thereby inhibiting NLRP3 inflammasome-mediated pyroptosis and alleviating CIRI through suppression of Nrf2-dependent NLRP3 inflammasome activation (Wang et al., 2025a). Beyond these pathways, additional targets of LIG in CIRI have been identified. LIG can upregulate erythropoietin expression and downregulate RTP801 via the ERK signaling pathway, thereby attenuating ischemia-reperfusion-induced neuronal injury (Wu et al., 2011). Meanwhile, heat shock protein 70 (HSP70) plays a crucial role in the neuroprotective effect. This component can induce the expression of protective HSP70 through activation of the MAPK pathway, thereby indirectly protecting against neuronal damage induced by oxygen-glucose deprivation-reoxygenation, and this process does not rely on the regulation of HSF1 (Yu et al., 2015).

## The therapeutic effects of LIG in transient ischaemic attacks

Transient ischemic attack (TIA) is defined as a transient episode of neurological dysfunction caused by focal ischemia of the brain, spinal cord, or retina, without evidence of acute infarction. As an important warning sign for stroke, TIA is widely recognized as a critical window for secondary prevention (Brisson et al., 2021). In studies investigating the protective effects of LIG against cerebral ischemic injury, experimental evidence from animal models has shown that oral administration of LIG (20 mg/kg or 80 mg/kg) at 2 h after ischemia significantly reduced infarct volume and alleviated brain edema in a dose-dependent manner. Moreover, LIG treatment markedly improved behavioral deficits in ischemic rats (Peng et al., 2007). Building upon these findings, further investigations by the same research group demonstrated that LIG exerts significant neuroprotective effects against brain injury induced by transient forebrain ischemia in rats. The underlying mechanisms are closely associated with its antioxidant and anti-apoptotic properties, providing important experimental evidence supporting the therapeutic potential of LIG in cerebral ischemic injury (Kuang et al., 2006).

## The therapeutic effects of LIG in intracerebral hemorrhage

Intracerebral hemorrhage (ICH), a common and severe neurological emergency, is caused by the rupture of cerebral blood vessels leading to the extravasation of blood into the brain parenchyma, which subsequently induces secondary brain injury. Clinically, ICH is characterized by sudden onset, rapid progression, and severe clinical manifestations, often resulting in varying degrees of neurological deficits, including limb paralysis and cognitive impairment. Notably, its mortality and disability rates are significantly higher than those of ischemic stroke (Gross et al., 2019). Li et al. reported the potential therapeutic effects of LIG in ICH. The results demonstrated that LIG exerted neuroprotective effects by inhibiting the Prx1/TLR4/NF-κB signaling pathway, thereby modulating immune responses and attenuating neuroinflammatory injury (Han et al., 2018). Subarachnoid hemorrhage (SAH) is another life-threatening subtype of stroke, accounting for approximately 3%–7% of all stroke cases. Relevant studies have shown that LIG can reduce brain edema, decrease blood-brain barrier permeability, and alleviate cerebral vasospasm. These effects subsequently reduced the number of apoptotic cells in the peri-injury brain tissue, suggesting that LIG exerts neuroprotective effects, at least in part, through the regulation of apoptosis (Chen et al., 2011).

## The therapeutic effects of LIG in geriatric traumatic brain injury

Geriatric traumatic brain injury (gTBI) has emerged as a major global public health concern requiring urgent attention. Epidemiological evidence indicates that the risk of mortality following traumatic brain injury increases significantly with age. Specifically, the 1-month mortality rate reaches 16.9% in patients aged 65–79 years and further rises to 31% in those aged ≥ 80 years, identifying advanced age as an independent risk factor for poor prognosis in gTBI (Lai et al., 2025). Recent advances in mechanistic studies have provided important insights into the potential therapeutic effects of LIG in gTBI. A recent study demonstrated that LIG promoted the phenotypic polarization of microglia in the injured brain from the pro-inflammatory M1 phenotype toward the anti-inflammatory, reparative M2 phenotype. This shift effectively suppressed the excessive release of pro-inflammatory cytokines, enhanced neuronal autophagy, and exerted neuroprotective effects. Consequently, LIG treatment significantly improved neurological deficits and alleviated post-traumatic memory impairment in aged TBI model mice, providing novel experimental evidence and a promising research direction for targeted therapeutic strategies in gTBI (Wu et al., 2025).

## The therapeutic effects of LIG in vascular dementia

Vascular dementia (VaD) is closely associated with chronic cerebral hypoperfusion, which progressively impairs neuronal structure and cognitive function (Inoue et al., 2023). Experimental studies have shown that LIG can alleviate cognitive dysfunction and brain injury induced by chronic ischemia, partly through enhancement of antioxidant capacity and improvement of cholinergic system activity (Kuang et al., 2008). Beyond its antioxidative effects, LIG also protects hippocampal neuronal structure and dendritic integrity by suppressing neuronal apoptosis and abnormal astrocyte proliferation in VaD models (Feng et al., 2012). Regulation of the AMPK/SIRT1 pathway appears to be another important mechanism underlying its neuroprotective effects. LIG reduces oxidative stress-induced neuronal injury and homocysteine accumulation while modulating apoptosis-related proteins, including Bax, cleaved caspase-3, and Bcl-2, thereby preserving neuronal function (Peng et al., 2022a). Endoplasmic reticulum stress is also involved in the progression of VaD. LIG reverses the abnormal activation of the BiP/p-IRE1α/XBP1s/CHOP pathway and restores SIRT1 and PDI expression, contributing to the attenuation of endoplasmic reticulum stress-mediated neuronal injury (Peng et al., 2022b). In addition, *Angelica sinensis* containing LIG has been reported to promote neurogenesis and neural stem cell proliferation through regulation of BDNF, p-CREB, and GABA expression, providing indirect support for the neuroprotective potential of LIG in VaD (Xin et al., 2013).

## The therapeutic effects of LIG in Alzheimer’s disease

Alzheimer’s disease (AD) is a progressive neurodegenerative disorder characterized by cognitive decline, synaptic dysfunction, and neuronal loss (Breijyeh et al., 2020). Multiple studies have shown that LIG exerts protective effects against AD through regulation of neuroinflammation, oxidative stress, mitochondrial dysfunction, autophagy, and amyloid-β (Aβ)-related pathology. In recent years, the multi-target and multi-pathway therapeutic potential of LIG in AD has become increasingly evident. Accumulating evidence demonstrates that LIG exerts comprehensive neuroprotective effects through precise modulation of key signaling pathways. LIG inhibits the abnormal activation of the TLR4/NF-κB pathway, thereby reducing the release of pro-inflammatory cytokines. It also attenuates endoplasmic reticulum stress-mediated apoptosis by regulating the GRP78/PERK/CHOP pathway, while promoting autophagic activity to facilitate the clearance of pathological substrates. In addition, LIG targets the PKA/AKAP1 signaling pathway to restore mitochondrial function, thereby alleviating neuronal dysfunction and cognitive deficits associated with AD (Zhang et al., 2025; Zhang et al., 2024a; Zhu et al., 2020; Li et al., 2015; Gao et al., 2021). In addition, LIG protects against Aβ-induced neurotoxicity through activation of the PI3K/Akt signaling pathway and inhibition of p38-related signaling (Xu et al., 2016). Aβ accumulation and impaired clearance are central events in AD pathology. LIG reduces cerebral Aβ deposition, improves synaptic structural integrity, and ameliorates memory impairment in APP/PS1 transgenic mice (Xu et al., 2018). In addition, Z-LIG suppresses Aβ25-35-induced neurotoxicity by modulating NF-κB signaling activity, accompanied by reduced expression of pro-inflammatory markers in the brain (Kuang et al., 2009). Regulation of Klotho-related signaling may also contribute to its anti-AD effects. LIG inhibits overactivation of the IGF-1/Akt/mTOR pathway, promotes Klotho expression and α-secretase activity, and enhances Aβ clearance, ultimately improving cognitive function (Kuang et al., 2017). Further evidence suggests that LIG attenuates oxidative stress, tau phosphorylation, and Aβ1–42 accumulation through modulation of Klotho/FoxO1-related signaling pathways (Katonova et al., 2025).

## The therapeutic effects of LIG in postoperative cognitive dysfunction

Postoperative cognitive dysfunction (POCD) is a common neurocognitive complication characterized by impairments in memory, attention, executive function, and information processing, particularly in elderly patients undergoing major surgery (Varpaei et al., 2024). Accumulating evidence suggests that neuroinflammation, oxidative stress, and impaired hippocampal neurogenesis contribute to the development of POCD (Zhang et al., 2024a). The hippocampus is highly vulnerable to surgical and anesthetic injury, and disruption of hippocampal neural stem cell activity is considered an important pathological feature of POCD. LIG has been shown to alleviate cognitive deficits in POCD models by promoting hippocampal neurogenesis and enhancing the proliferation and differentiation of neural stem cells. These effects were accompanied by increased expression of synaptic plasticity-related proteins, including Syp, PSD-95, GAP-43, Syn-IIa, and ERK1/2, suggesting improved neuronal repair and synaptic function (Sun et al., 2021). These findings indicate that LIG may ameliorate POCD by promoting hippocampal neurogenesis and restoring synaptic plasticity, highlighting its potential as a neuroprotective agent for perioperative cognitive impairment.

## The therapeutic effects of LIG in Parkinson’s disease

Parkinson’s disease (PD) is a progressive neurodegenerative disorder characterized by dopaminergic neuronal loss in the substantia nigra and subsequent motor dysfunction (Hayes, 2019; Pagano et al., 2024). Neuroinflammation, oxidative stress, mitochondrial dysfunction, and abnormal microglial activation are closely involved in PD progression (Rocha et al., 2018). Evidence indicates that LIG markedly improves motor dysfunction in PD models and attenuates the selective loss of dopaminergic neurons in the substantia nigra pars compacta. These effects are closely associated with activation of endogenous antioxidant defense systems and inhibition of oxidative stress-induced microglial overactivation and apoptosis. In addition, LIG precisely regulates microglial phenotypic polarization by promoting the transition from the pro-inflammatory M1 phenotype to the anti-inflammatory M2 phenotype. This shift is accompanied by activation of the Nrf2-TrxR signaling pathway, which restores redox homeostasis and mitigates the vicious cycle between oxidative stress and neuroinflammation. Collectively, these findings highlight the multi-target and synergistic advantages of LIG in modulating PD pathophysiology, providing important experimental evidence and supporting the potential of LIG as a promising candidate for the development of novel therapeutic strategies for PD (Peng et al., 2024).

## The therapeutic effects of LIG in glioblastoma multiforme

Glioblastoma multiforme (GBM) is the most common and aggressive primary malignant tumor of the central nervous system and is characterized by rapid proliferation, strong invasiveness, and poor prognosis (Sang et al., 2021; Ohgaki et al., 2013). Recent studies have suggested that LIG may exert anti-GBM effects through regulation of tumor cell migration and enhancement of drug delivery efficiency. LIG inhibits the migratory ability of T98G glioblastoma cells by regulating cytoskeleton-associated signaling molecules, including RhoA, Cdc42, and Rac1, thereby suppressing the invasive behavior of GBM cells (Yin et al., 2013). In addition, LIG has been reported to enhance blood-brain barrier permeability and reduce P-glycoprotein-mediated drug efflux, which may increase intracerebral accumulation of temozolomide (TMZ) and improve its therapeutic efficacy. Based on these properties, a PLGA-mPEG nanoparticle co-delivery system encapsulating both TMZ and LIG was developed and showed enhanced anti-glioblastoma effects in experimental studies (Ke et al., 2025).

## The therapeutic effects of LIG in atherosclerosis

Atherosclerosis (AS) is a chronic vascular disease characterized by lipid accumulation, oxidative stress, endothelial dysfunction, and persistent inflammation within the arterial wall, and serves as an important pathological basis for multiple cardiovascular and cerebrovascular disorders (Baumer et al., 2025; Glass et al., 2001). Increasing evidence also supports a close interaction between vascular dysfunction and neurological diseases through the heart-brain axis (Simats et al., 2025; Bolaji et al., 2025). LIG has shown protective effects against AS through antioxidative, anti-inflammatory, and lipid-regulating mechanisms. Activation of the NRF2/ARE signaling pathway enhances antioxidant defense capacity, promotes nuclear accumulation of NRF2, and attenuates oxidative stress-induced endothelial injury, thereby reducing lipid peroxidation and atherosclerotic plaque formation in experimental models (Zhu et al., 2019). Beyond NRF2/ARE-mediated antioxidative effects, the gut microbiota has emerged as a critical regulator in the pathogenesis of AS. Dysbiosis of the gut microbiota contributes to impaired intestinal barrier function, chronic low-grade inflammation, and metabolic disturbances, thereby accelerating atherosclerotic progression (Strasser et al., 2021; Vaiserman et al., 2017). In addition, LIG improves lipid metabolism, suppresses inflammatory responses, and preserves intestinal barrier integrity through modulation of gut microbiota-related pathways and cannabinoid receptor 2 signaling, ultimately contributing to attenuation of AS progression (Liu et al., 2024).

## The therapeutic effects of LIG in autism spectrum disorder

Autism spectrum disorder (ASD) is a neurodevelopmental disorder characterized by impaired social communication and repetitive behavioral patterns (Hirota et al., 2023; LORD et al., 2018). Currently, most clinical interventions for ASD remain symptomatic, primarily focusing on improving social deficits and repetitive behaviors, while lacking effective strategies that target the underlying disease mechanisms. Therefore, the identification of safe and effective therapeutic agents capable of modulating disease progression is of urgent importance (Sharma et al., 2018). Its pathogenesis is complex and involves multiple factors, including neuroinflammation, immune dysregulation, and abnormal neural development (Bakke, 2022; Sacai et al., 2020). Abnormal cerebral blood perfusion has also been implicated in ASD-related brain dysfunction (Zilbovicius et al., 2000). Recent studies have suggested that LIG may alleviate autism-like behaviors through regulation of ferritinophagy-related pathways. In VPA-induced and BTBR mouse models of ASD, LIG inhibited excessive ferritinophagy in cerebellar Purkinje cells by modulating the ULK1/NCOA4 signaling pathway, thereby improving social interaction deficits and repetitive behaviors (Zhou et al., 2024).

## The therapeutic effects of LIG in demyelination

Demyelination is an important pathological feature of ischemic brain injury and contributes to impaired neural signal transmission and neurological dysfunction following stroke (Vagionitis et al., 2024). Increasing evidence suggests that LIG exerts protective effects against demyelination through regulation of neuroinflammation and myelin repair-related pathways. In distal middle cerebral artery occlusion (dMCAO) models, LIG significantly reduced infarct volume, improved neurological function, and attenuated demyelinating lesions in the brain. These effects were accompanied by increased expression of myelin basic protein (MBP), brain-derived neurotrophic factor (BDNF), and glial fibrillary acidic protein (GFAP), indicating enhanced myelin repair and neurotrophic support. At the same time, LIG suppressed neuroinflammation by reducing the expression of inflammatory mediators, including TNF-α, IL-6, IL-17A, AIM2, caspase-1, ASC, and IBA1, suggesting inhibition of AIM2 inflammasome-related signaling pathways (Liang et al., 2025).

## The therapeutic effects of LIG in anxiety disorders

Anxiety disorders are common psychiatric conditions characterized by excessive fear, anxiety, and autonomic dysfunction, often accompanied by sleep disturbance and cognitive impairment (Leichsenring et al., 2023). The increasing prevalence of anxiety disorders has been closely associated with chronic psychological stress and environmental factors (DeGeorge et al., 2022). Recent studies have identified artificial light at night (ALAN) exposure as a potential contributor to anxiety-like behaviors through disruption of mitochondrial homeostasis and neuronal function. In experimental models, LIG significantly alleviated ALAN-induced anxiety-like behaviors by regulating mitophagy and PI3K/Akt-related signaling pathways. These effects were associated with preservation of neuronal ultrastructure, improvement of synaptic integrity, and attenuation of neuroinflammatory injury (Gao et al., 2025).

## Shared and disease-specific mechanisms of LIG across CNS disorders

**FIGURE 3:**
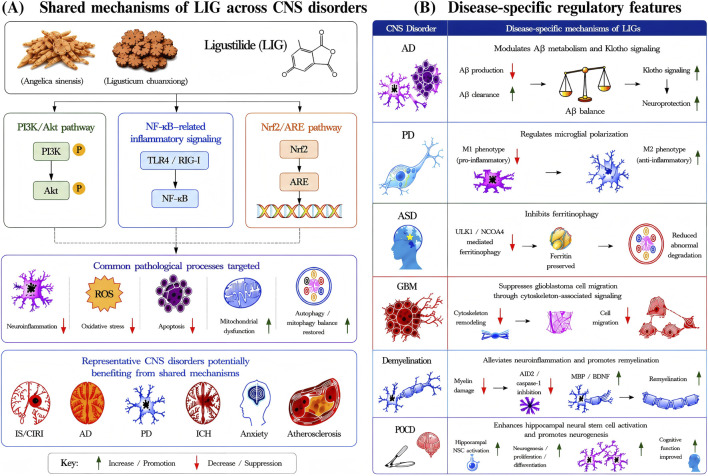
Shared and disease-specific mechanisms of LIG across CNS disorders. **(A)** Shared mechanisms of LIG across CNS disorders. **(B)** Disease-specific regulatory features.

Despite the heterogeneity of CNS disorders, oxidative stress, neuroinflammation, mitochondrial dysfunction, apoptosis, and impaired autophagy are common pathological processes involved in many of these diseases. Accordingly, the neuroprotective effects of LIG across different CNS disorders are mainly associated with regulation of PI3K/Akt, NF-κB, and Nrf2-related signaling pathways. In addition to these shared mechanisms, several disease-specific effects of LIG have also been identified. LIG regulates Aβ metabolism and Klotho-related signaling in Alzheimer’s disease, modulates microglial polarization in Parkinson’s disease, inhibits ferritinophagy in autism spectrum disorder, suppresses glioblastoma cell migration, and promotes myelin repair in demyelinating injury. The shared and disease-specific mechanisms of LIG across CNS disorders are summarized in Figure 3.

## Preclinical and clinical applications

LIG has demonstrated broad neuroprotective effects in multiple experimental models of CNS disorders, including ischemic stroke, Alzheimer’s disease, Parkinson’s disease, vascular dementia, and traumatic brain injury. Its pharmacological activities are mainly associated with anti-inflammatory, antioxidant, anti-apoptotic, and mitochondrial-protective mechanisms. Importantly, LIG is capable of penetrating the blood-brain barrier, providing a pharmacokinetic basis for its application in CNS disorders. Current preclinical evidence also suggests relatively favorable biocompatibility and safety under conventional experimental conditions. Despite these promising findings, the therapeutic potential of LIG remains largely limited to preclinical studies, and well-designed clinical investigations are still lacking. Further studies are needed to evaluate its long-term safety, optimal administration strategies, and clinical efficacy in different CNS disorders.

## Translational challenges and strategies for LIG

Despite these advances, the clinical translation of LIG remains challenging. Most existing evidence is derived from preclinical models, whereas clinical studies evaluating the efficacy and safety of LIG are still lacking. Moreover, issues including poor chemical stability, low oral bioavailability, rapid metabolism, and insufficient long-term safety evaluation have not yet been fully resolved. Although several novel delivery systems have shown encouraging results in experimental studies, their clinical applicability, large-scale production feasibility, and long-term biosafety still require further validation.

## Conclusion

Despite the encouraging neuroprotective effects of LIG across multiple CNS disorders, several limitations should be acknowledged. First, most currently available evidence is derived from rodent models or immortalized cell lines, which may not fully reproduce the pathological heterogeneity and complexity of human CNS disorders. In addition, substantial differences in experimental models, dosing regimens, administration routes, and outcome assessments limit the comparability and reproducibility of current findings. Notably, several signaling pathways, including PI3K/Akt, NF-κB, Nrf2/ARE, and AMPK, were repeatedly identified across different studies, suggesting that some observed mechanisms may represent convergent downstream responses rather than disease-specific regulatory targets. Furthermore, most published studies reported positive findings, whereas contradictory or negative results remain limited, potentially leading to overestimation of the therapeutic potential of LIG. Most importantly, clinical evidence remains absent, and challenges including poor chemical stability, low oral bioavailability, and insufficient long-term safety evaluation continue to hinder translational application. Therefore, future studies should emphasize standardized experimental protocols, mechanistic integration, and clinical validation to better define the therapeutic value of LIG in CNS disorders.
